# Hydrophobia

**Published:** 1879-11

**Authors:** John J. Caldwell

**Affiliations:** Baltimore


### HYDROPHOBIA.

BY JOHN J. CALDWELL, M. D., BALTIMORE.

Mr. Youatt's description has been the one most uniformly accepted and quoted. He says: "The disease manifests itself under two forms: the furious form, characterized by augmented activity of the sensorial and locomotive systems, a disposition to bite, and a continual peculiar bark. The animal becomes altered in habits and disposition, has an inclination to lick or carry inedible substances, is restless, and snaps in the air, but is still obedient and attached. Soon there are loss of appetite and the presence of thirst, the mouth and tongue swollen; the eyes red, dull, and half closed; the skin of the forehead wrinkled; the coat rough and staring; the gait unsteady and staggering; there is a periodic disposition to bite; the animal in approaching is often quiet and friendly, and then snaps; latterly, there is paralysis of the extremities, the breathing and deglutition be come affected by spasms; the external surface irritable, and the sensorial functions increased in activity and perverted, convulsions may occur. These symptoms are paroxysmal, they remit and intermit, and are often excited by sight, hearing and touch.

"The sullen form is characterized by shyness and depression, in which there is no disposition to bite and no fear of fluids. The dog appears to be unusually quiet, is melancholy, and has no depression of spirits; although he has no fear of water he does not drink; he makes no attempt to bite, and seems haggard and suspicious, avoiding society and refusing food. The breathing is labored, and the bark is harsh, rough, and altered in tone; the mouth is open from the dropping of the jaw; the tongue protrudes, and the saliva is constantly flowing. The breathing soon becomes more difficult and laborious; there are tremors, and vomiting, and convulsions."

"As regards the diagnosis, there is no particular symptom which can be relied upon as forming a distinguishing mark of this disease, the symptoms must be taken collectively, and then there is no disease similating it; thus the peculiar combination of disturbance of the muscles of the pharynx and larynx, the psychical hyperæsthesia and fear, the altered voice, the difficult breathing and swallowing, the perverted appetite, the hallucination, and the easily excited rage are for the most part the diagnostic signs. The dread of water, the almost characteristic symptom in man, is not always present, as may be proved in recorded cases. On the other hand, this symptom may be met with in other diseases, thus in hysteria, we may have what is called hysterical hydrophobia, where the sight of water induces a paroxysm of hysteria, but this only lasts a short time."

The few remarks I shall make, following the above observations upon this mysterious and almost unknown disease, will have for their object, to call the attention of the reader to,

First.—The terminology.

Second.—The analogy existing between this and other incubative poisons.

Third.—To some points of treatment.

Fourth.—To refer to a few cases under our own observation.

It would be well if the profession could adopt another name which would have a better and closer relation to the pathology and symptoms of this malady. As *toxæmia canina vel felina*, or the poisonous effects following the bite of rabid dogs or cats.

This poison has its inception, its incubation, and its explosion or final demonstration, as well as its point or points of election.

The same may be said of the bite of venomous serpents or insects, which either act locally or overwhelm the nervous system; also of the poison of small pox and scarlet fever, which select the surfaces; of puerperal fever, electing the peritoneum; of typhoid poisoning, electing the mucous membrane of the bowels; or spotted fever, electing the meninges of the brain and spinal cord. Thus the poison, commonly known as rabies virus, no matter where inserted, elects for its point of explosion the brain, the upper portion of the spinal cord, the muscles of the neck and chest; while strychnine and tetanus, choose the spinal axis, leaving the brain free. In hydrophobia, the brain, the medulla, and the cervical spine are, in the last stages, more or less hyperæmic, hence the proneness to hyperæsthesia and to frequent clonic spasm, and the irritation of light, of sound, of draughts of air, of particles of fluids, or solids, upon the muscles of deglutition. Indeed, so hyperæsthetic are these centers that the slightest irritation will produce convulsion. The pathology of this disease is involved in great obscurity; indeed, the same may be said of all incubating poisons acting by reflex forces upon distant centers, in consequence of minuteness of nerve vesicles, requiring the aid of powerful microscopic observation to witness these minute changes, as well as the spectrum to appreciate the chemical and vital metamorphosis. None of our Savans have been able to trace the *Materies morbi*, or its pathological tracts from the point of inception to their field of explosion in any of these incubative poisons. But what particularly maintains with the poison under discussion; what especially distinguishes it from the others, is its election of three of the most vital points of the economy; viz: The brain appendages, the respiratory and circulatory centers, while other incubating and elective diseases involve more remote and less vital localities. Here, we believe, rests the explanation of the great dread of, and fearful mortality from hydrophobia, of tetanus and poison of strychnia, but especially with the first named, for the brain, and its membranes and the upper part of the spinal cord, and sometimes the nerves of the wound, the mouth, the windpipe, and the lungs, are found in a congested state, no doubt principally from the loss of function, and thus we can account for the unhappy symptoms of pain, and uneasiness in the throat and lungs, during the last scene or stage of the disease, for the stiff and unweildy tongue, for the indistinct speech, for the rigid muscles of the throat, for the impeded deglutition, the dread of swallowing, and the convulsious of the muscles of this locality, and for the irregular mental disturbances. The causes have been generally attributed to rabid cats and dogs, but later writers have attributed the disease to several other wild and domestic animals, while laboring under the various passions, such as anger, rage, freight, heat, etc. A late writer has claimed the disease uniformly resulting from the bite of a pole-cat or skunk. In an article upon the subject, (we think in the *Virginia Medical Monthly* for September), is given a large experience, and much data from the field of practice of the writer in Nevada. In our own experience of four cases, (three while a student in Bellevue, and one in private practice), one that proved rapidly fatal, was caused by the bite of a cat, while the pound master was endeavoring to rescue her from a number of dogs in the pound.

Again, we read, from the *Evening News*, of a remarkable case of hydrophobia:

Chicago, September 16th.—A singular case of death from hydrophobia occurred here last Saturday. The facts of the case have just been made public, and are as follows: Charles Haake, a boy six years of age, was bitten on the head by a cat, which he was chastising for some reason, about four weeks ago. The wound was so slight that the child did not complain of it at all, but on Saturday morning last, he was taken with all the symptoms of hydrophobia, and died before night. The cat has never at any time exhibited any signs of madness, and the physicians who attended the boy regard it as the most remarkable case extant.

From the *Gaz. des Hopitaux*: Two Russian physicians recently treated a young girl twelve years old who had been bitten through the skin by a mad dog. The wound had been immediately cauterized with lunar caustic and cicatrization was complete in eight days. Three days later the patient was attacked with diphtheria, and paralytic aphonia. Seventeen days after the bite she was seized with convulsions. The immediate inhalation of three cubic feet of oxygen was practiced with such effect that in two hours and a half, the patient was resting quietly and comfortably. Two days later the same symptoms supervening, the inhalation was again practiced for forty-five minutes when the convulsions ceased. Monobromide of camphor was prescribed for three weeks on account of a slight dyspnœa. A month later there was a slight atony of the muscles of the leg, but these symptoms gradually passed away, leaving no trace of the disease except a slight aphonia due to the effect of the diphtheria. In 1875 Drs. Paul and Josias treated a similar case with this same method, but the patient died, although the spasms were allayed temporarily by the oxygen.

From the *Boston Medical and Surgical Journal*, March 7th 1878:

If hydrophobia is a dreadful disease for the patient, it is also such for the physician and attendants; even for those, if any there still be, who regard the phenomena as of a purely psychical origin. It is perhaps the disease of all others in which the physician feels himself not only powerless to cure, but often even to alleviate suffering. Morphia and ether, those invaluable aids in most hopeless and painful diseases, betray us here. All anti-spasmodics and sedatives and evacuants have been tried, from injections of tobacco, and bleeding, forty years ago, to steam baths, chloral and bromide latterly, with uncertain and unsatisfactory results, until one is almost led to think that the resort to the pillow, at one time prevalent in parts of Europe, if not an ethical was at least not a cruel procedure. Jaborandi, or its alkaloid pilocarpine, has been of late suggested as a remedy by elimination, with the same end in view as the treatment by steam baths, known as Duisson's system; but we know of no cases where it has been tried, and should not anticipate any degree of success.

At least three trustworthy cases have been reported within late years in which recovery from symptoms similar to those of hydrophobia has followed the use of curara. Was the disease in each or any of these hydrophobia? Was the recovery due to curara? Certainly none of the three is so convincing as to disarm skepticism, though the case reported by Dr. Watson, of Jersey City,* is striking. The amount of curara used was very small, about one-third of a grain, and was ad ministered subcutaneously at intervals of three hours, the first injection containing one-sixteenth of a grain, the second one-ninth of a grain, and the third one-sixth of a grain. No physiological effects are reported to have been produced. It was admistered on the sixth day of the disease, when marked symptoms of exhaustion are said to have been present. In case of Dr. Offenberg† morphia and chloroform having been used without success, three grains of curara were introduced within five and a half hours in seven injectious. Muscular paralysis supervened, reaching its maximum on the second day. The second day after the injections a slight relapse of the first symptoms was controlld by one half a grain of curara. The injections were begun three hours after the outbreak of the symptoms. The recovery was slow. In the third case, that of Dr. Polli. the subject was a child twelve years of age; twenty centigrammes (three grains) of curara were administered in five and a half hours in seven injections. Partial paralysis was induced. A return of the previous symptoms

*Amer. Jour. of the Med. Sciences, July, 1876.

†Dentsches Zeitschrift fur Praktische Medicin No. 52.

three days later was controlled by three centigrammes (half a grain).

On the other hand, we have at least three cases in which curara was used with no effect, or at least unsatisfactory one. In one case* six and a half grains, were given in four injections at intervals of two hours; the first injection containing one grain, the second one grain, the third two grains, the fourth two and a half grains. No effect was produced. In the case reported in the *Journal* May 17, 1879, four grains were injected in half-grain doses within twenty one hours, but on this occasion morphia, nitrite of amyl, chloroform, and chloral were also used.

In a late case, not yet reported, which terminated fatally, four and a third grains were injected in varying doses within five and a half hours. Other reports exist of the successful use of curara, but in most the amount was extremely small.

The great present drawback to the use of curara as a drug is its very uncertain purity, scarcely any two specimens being the same in this particular. We may thus account in part, for the different results obtained in those cases where it has been tried, and the unsatisfactory impression they make. The character of the effect produced is always the same, but the amount varies with the quantity of extractive matter. Apart from any experience, ought we to expect good results from what we know of the physiological action of curara? We learn from Claude Bernard's† experiments that it produces some peculiar effect upon the terminations of the motor nerves, "unhooking," to use his expression, first the central terminations in the cord, and subsequently the peripheral terminations in the muscles; this latter is only reached where the circulation is carried on for a longer time, as in cold-blood animals, or with artificial respiration in mammals. The result is an inability of the motor nerves to cause contraction of the muscles. The muscles of the extremities are apparently the first cut off, then those of respiration, and finally the heart.

*Lancet 1872 Page 257.

†Physiologie generale, 1872, page 237.

Death in hydrophobia usually takes place from asthenia, the result of constantly recurring and exhausting convulsions, or from asphyxia during one of these spasms. From what late investigations have taught us of the minute pathology of the nerve centres in the medulla and at the base of the brain we may attribute the spasmodic manifestations to the congestion and hyperæsthesia of these centres, and of parts of the cord in a less degree. Curara can produce no effect upon these central lesions. An irritating impression is received, is transmitted, is registered at the central office, producing there the proper commotion; here, however the routine is broken; orders may be prepared for the muscles, but they can not be sent, and do not arrive.

All we can expect, then, of curara is an avoidence of the outward and visible sign of spasm. This is not much, but we are not ready to say that it may not be enough in some cases to prolong life and give an opportunity for the elimination of the hydrophobic poison.

Certain precautions should be borne in mind. The efficacy of any specimen should be previously ascertained by experiment. It should only be administered hypodermically. In preparing it the drug should be reduced to a fine powder, diluted with a little distilled water, and filtered through filtering paper. This should be done at least on the day or better still at the time of use, as a second precipitate of extractive matters is formed upon standing which cause abscesses at the points of injection. Larger quantities should be administered than has hitherto been usually the case, either in the form of one large dose, or, perhaps better, of frequent small doses. One to two decigrammes (about one and a half to three grains) have been given in tetanus* The earlier in the disease the drug was used the more favorable result we should expect, and the less the quantity of curara we should suppose necessary.

We can not but think it worth while to make further use of curara in hydrophobia in view of the results so far obtained, and certainly in the absence of anything more promising.

*Bo is in, Dict. de Medicin et de chirurgiel Pratiques.

I may further say as to treatment. As to prophylaxis; it is the uniform and established plan, by most practitioners, to cauterize and wash the wound as soon as practicable, the sooner the better. Indeed, if this mode should maintain throughout the non-professional, as well as the professional world, many valuable lives might be saved. We would suggest that this, as well as many other medical truths, should universally be taught and practiced, as is the case among the barbaric and semi-barbaric races.

We will here cite a case occurring some ten or twelve years ago, wherein a little girl was badly bitten by a rabid dog. It so happened, while visiting one of the suburban villages of New York City, her grandfather sent her to the village post office with a pony-cart and driver. While waiting at the store door, a dog came running along snapping at everything in his way. He bit her pony through the nose. During the struggle she was thrown to the ground, the dog biting her through the pectoral muscles of the right side, incising, lacerating, scratching and bruising the parts in a terrible manner. A few minutes after this accident we were by her side. We washed the parts thoroughly and canterized them with nitro-muriatic acid. Our patient recovered, and at our last hearing was doing well, enjoying the best of health (several years after the accident) while the pony and other animals bitten by this dog suffered and died with this disease within a few weeks.

INCUBATION.

If during the stage of incubation an agent could be introduced by the mouth or rectum which would have the power of acting as an antidote, and thus neutralize the poison which is circulating in the blood, and prevent its acting perniciously upon the nervous centers and especially the brain and medulla spinalis, the discoverer would confer a boon upon humanity and place himself in the temple of fame side by side with the immortal Jenner.

REMEDIES.

Of all remedies, none are so fully adapted to the symptoms as chloroform, chloral hydrate, and the crotchloral hydrate; to allay and lessen the hyperæmia and hyperæsthesia of the neural tripod of life, the brain, medulla spinalis, and great sympathetic, in order to maintain life's currents and facilitate the molecular change, and thus eradicate the poison, the constant galvanic current should be exhibited powerfully and continuously through these implicated axes. If necessary, they should be continued for a number of days. Nourishment should be administered by the rectum, and hypodermically.

In conclusion, we will cite the following to corroborate our theory.

Hammond, page 550: "Dr. Schward, of Milan, attempted the cure of this disease by the primary galvanic current. In one case the current was feeble, and was continued for nineteen hours. Great improvement ensued, the oppression disappeared, and the dysphagia was entirely relieved. Through some misunderstanding advantage was not taken of these ameliorations, and the patient was allowed to die. In the other case, which was one of undoubted hydrophobia, occurring in a girl nine years old, the current from twenty-two Daniel's cells was employed. The current was passed from the soles of the feet to the forehead, for fifty-eight hours, almost continuously, and the durations of the disease prolonged to seven days and seven hours, when the patient died. During the last two days there were no hydrophobic symptoms.

